# A scoping review of psychiatric conditions associated with chronic pain in the homeless and marginally housed population

**DOI:** 10.3389/fpain.2023.1020038

**Published:** 2023-04-28

**Authors:** Kathryn Rintoul, Esther Song, Rachel McLellan-Carich, Elizabeth N. R. Schjelderup, Alasdair M. Barr

**Affiliations:** ^1^ Department of Anesthesiology, Pharmacology & Therapeutics, Faculty of Medicine, University of British Columbia (UBC), Vancouver, BC, Canada; ^2^ British Columbia Mental Health and Substance Use Services Research Institute, Vancouver, BC, Canada; ^3^ Department of Psychiatry, Faculty of Medicine, UBC, Vancouver, BC, Canada

**Keywords:** chronic pain, mental illness, psychiatric morbidity, homeless, systematic reviews

## Abstract

The present review sought to examine and summarise the unique experience of concurrent pain and psychiatric conditions, that is often neglected, within the population of homeless individuals. Furthermore, the review examined factors that work to aggravate pain and those that have been shown to improve pain management. Electronic databases (MEDLINE, EMBASE, psycINFO, and Web of Science) and the grey literature (Google Scholar) were searched. Two reviewers independently screened and assessed all literature. The PHO MetaQAT was used to appraise quality of all studies included. Fifty-seven studies were included in this scoping review, with most of the research being based in the United States of America. Several interacting factors were found to exacerbate reported pain, as well as severely affect other crucial aspects of life that correlate directly with health, within the homeless population. Notable factors included drug use as a coping mechanism for pain, as well as opioid use preceding pain; financial issues; transportation problems; stigma; and various psychiatric disorders, such as post-traumatic stress disorder, depression, and anxiety. Important pain management strategies included cannabis use, Accelerated Resolution Therapy for treating trauma, and acupuncture. The homeless population experiences multiple barriers which work to further impact their experience with pain and psychiatric conditions. Psychiatric conditions impact pain experience and can work to intensify already adverse health circumstances of homeless individuals.

## Background

Homelessness is currently a significant public health issue in urban centers worldwide, and has increased recently in regions such as the West Coast of North America (1, 2). More recent data indicate that in the United States, approximately half a million people experience homelessness on a single night (1), while in Canada, more than 235,000 people experience homelessness in a given year (2). Based on this key issue, it remains a high priority for research studies to continue to identify the various factors that act to disadvantage the homeless population. In particular, the topic of pain with comorbid psychiatric conditions within the homeless population is still notably understudied, and there is an urgent need to better understand this complex biopsychosocial issue and how it effects related individuals (3, 4). Importantly, research should continue to advance and better elucidate how pain is affecting this at-risk population, as this is crucial to understand how to better address issues within the various levels of the healthcare system. It is already evident that there are a number of factors that impede homeless individuals from seeking help for pain-related conditions, including the manner in which healthcare professionals behave towards them, as well as reliable access to high-quality clinical staff who have a deeper understanding of the complexities of multimorbid mental illness (5–7).

Homelessness and precarious housing are often intertwined with various mental health disorders at a rate substantially greater than the general population (8–12). The frequency and severity of major mental illnesses are alarming, including conditions such as anxiety, major depressive disorder, post-traumatic stress disorder (PTSD), psychosis, bipolar disorder, substance use disorders, and many more (13). Trauma is relatively common in the homeless population (8), often occurring early in life and thus not a direct consequence of living in homeless conditions; this early trauma can therefore create a psychological vulnerability to develop subsequent mental health issues when facing the severe stressors of “life on the street” (14). Impaired mental health - on its own—creates challenges and unequal opportunities for a host of various issues pertaining to housing, employment, seeking out healthcare, and proper nutrition (7, 15–17). In addition, both psychiatric conditions (such as depression) and pain can exacerbate disadvantageous health and further feed into a negative feedback loop that creates worse health outcomes along with decreased functioning in daily life. Chronic pain is often substantial and untreated within the homeless population, and its impact can be exacerbated within a large subset of individuals experiencing psychiatric symptoms (18). Stigma and biases towards the homeless population, including by those involved with their healthcare- can have a grave effect on their treatment, potentially aiding in the development of pain (18–20). Experiencing homelessness makes acquiring health services onerous. Some examples of how this inequity manifest include the following: difficulties in obtaining a health card due to the lack of a permanent address; unable to afford necessary fees associated in treatment; making appointments *via* phone call; disconcerting appearance; difficulties with maintaining a physician who has the individual's extensive medical records and is able to provide consistent treatment (21–23). All these barriers then lead to the overutilization of emergency health resources (24).

The aim of the current article therefore was to (a) summarize and (b) highlight the key points and “takeaway” messages of the literature on homeless or precariously housed individuals with reported chronic pain and concurrent psychiatric conditions; additionally, (c) we also sought to summarized this information comprehensively in a table where readers can easily access the key points of these studies.

We hypothesized that the homeless or precariously housed individuals with concurrent psychiatric issues are (a) at a higher risk of having worsening of health-related issues due to the snowball effect from intertwined and complicated relationship between psychiatric illnesses, day-to-day issues including living and financial status, and pain. And for this reason, we (b) further hypothesized that this cohort would respond better in treatments that encompass the variables that simultaneously contribute to the exacerbation of health-related issues than the commonly used traditional therapies such as medication therapy and opioid replacement therapy.

## Materials and methods

A scoping review was conducted on associated psychiatric conditions with reporting pain in the homeless population. The review was registered on the PROSPERO: International Prospective Register of Systematic Reviews [CRD42021268224]. See PRISMA checklist. While the current review did not fully satisfy the requirements PRISMA requirements for a systematic review, it incorporated many of these features to strengthen the comprehensiveness and objectivity of the review.

We approached the electronic search of articles published at any time using MEDLINE, EMBASE, psycINFO, and Web of Science, using the key words that are listed below.

1. Keywords: homeless persons/ or homeless youth/(homeless or unhoused or housing insecure).mp. [mp = title, abstract, original title, name of substance word, subject heading word, floating sub-heading word, keyword heading word, organism supplementary concept word, protocol supplementary concept word, rare disease supplementary concept word, unique identifier, synonyms]
  1 or 2
2. Keywords: exp Pain/
  (pain or painful or pains).mp. [mp = title, abstract, original title, name of substance word, subject heading word, floating sub-heading word, keyword heading word, organism supplementary concept word, protocol supplementary concept word, rare disease supplementary concept word, unique identifier, synonyms]
  4 or 5
3. Keywords: exp Mental Disorders/
  (psychological disorder or psychiatric disorder or mental illness or mental health conditions).mp. [mp = title, abstract, original title, name of substance word, subject heading word, floating sub-heading word, keyword heading word, organism supplementary concept word, protocol supplementary concept word, rare disease supplementary concept word, unique identifier, synonyms]
  7 or 8
4. Boolean operator: 3 and 6 and 9
5. Limits Language: English language
6. Limits date: N/A
7. Limits subjects of studies: N/A
8. Selection: Removal of duplicate articles and manual disposal of articles that do not fit the criteria.

In addition, we found grey literature through utilizing google scholar and employing the keywords: homelessness, pain, and mental disorders.

We extracted articles using these search terms after consulting with a subject librarian, as conducted previously (25–27). In addition, we used search terms from published articles examining similar discourse to gain more insight. Studies were included if the article was:

1. Published in a peer-reviewed journal.
2. The study was empirical in nature.
3. It examined participants who were homeless, using Gaetz et al.'s definition (28), defined as peoples, on a spectrum, that on one end have unstable and inappropriate housing and those who are without shelter; studies that examined psychiatric or mental disorders; and investigated pain. For this review, we define homelessness using Gaetz et al.'s definition (28) which encompasses homelessness as those who fall into four categories.
  a. The first being those who can be considered unsheltered, or inhabiting public or private places with no consent or contract, or those occupying a space that in not intended to be a long-term place.
  b. Secondly, those who live in an emergency shelter, which can be defined as those in overnight shelters, those suffering from family violence, or people escaping from arrangements due to disaster or demolition such as fires, flooding, etc.
  c. Those who are provisionally accommodated, meaning those who have accommodations without security or arrangements that are temporary.
  d. Finally, those who are risk of homelessness, which includes factors such as insecure employment, unexpected unemployment, soon to be discontinued supported housing, risk of eviction, critical and continued levels of mental illness, dissolution of a household, abuse in one's current household, or institutional care that is insufficient or inappropriate.

We excluded studies that examined the history of a person or populations homelessness, who at the time of the study, were no longer homeless. Additionally, as a team, we made the decision to further exclude any case-studies, as the results from those were not as useful for our questions.

Based on the inclusion criteria of both the population of homeless adults and youth and the domain of pain and concurrent psychiatric disorders, each database was searched by the first reviewer (KR). The articles, retrieved from the respective databases, produced abstracts and titles, which were subsequently exported into Covidence. Abstracts and titles were then screened by two reviewers (KR, ES) and assessed for fitness of abstracts, based on the inclusion criteria set out. Both reviewers (KR, ES) then read the full articles thoroughly and excluded those that had focused on excluded parameters, examining the full articles for fitness, looking at factors such as study population, mean age of participants, the aim of the study, the study results, and conclusions drawn from results. Due to the nature of our review, and not wanting to exclude data, we initially did not place limitations on study design, and therefore, needed to find a more appropriate appraisal tool to assess quality of all the included study designs. Which after discussion with a subject librarian, we decided to use the PHO MetaQAT. The PHO MetaQAT is a more thorough quality appraisal tool that favours qualitative research and more easily addresses the relevancy, reliability, validity, and applicability for public health research. Covidence extracted data was then added to an excel spreadsheet to use the PHO MetaQAT, to assess quality of all included studies. Discussions surrounding what data points were appropriate for further examination were done *via* a conversation with one reviewer (KR) and the corresponding author (AB). Once we had a clear agreement as to what exactly we were looking for, two reviewers (KR, EMK), set out to examine the excel spreadsheet to further extract specific data of importance. If a disagreement came about, EMK and KR would both discuss their reasonings behind a recommendation and decisions were made between the reviewers. In rare circumstances, if the two reviewers could not agree, decisions were made by the corresponding author (AB). After discussion, we further excluded 3 studies which utilized a case-study method, due to the data not being generalized. The results from included studies were reported narratively.

## Results

### Study characteristics

There were 57 studies included that were published over 28 years (1993–2021) (Table 1). Of the 57 studies identified, individual sample sizes ranged from 12 in Flanagan et al. (29) to 1,018,741 in Gundlapalli et al. (30). Among them, the great majority were from the United States with 41, 8 from Canada, 4 from the United Kingdom, 2 from Japan, and lastly, 1 was from France and 1 was conducted in Australia.

**Table 1:** Prevalence of pain in published research on pain, mental health, and homelessness.

Study ID	Country in which the study conducted	Study design	Population description	Types of pain	Location of pain mentioned in paper	Methods of how pain was examined	Total number of participants
Amato 2019	United States	Cohort Study	Individuals who were homeless at the time of their interaction with the ED. Individuals who were enrolled in Medicaid and not identified as homeless.	Not specified.	Abdominal pain, chest pain, back pain, headache, leg pain, and toe pain.	Electronic medical reviews including chief complaints, where the chief complaints were categorized by mental health, medical and trauma complaints, or conditions.	986 (homeless), 3,482 (Medicaid); total of 4,468
Bassuk 2001	United States	Cross sectional study	Women in the Worcester Family Research Project, where almost half were sheltered homeless and other half extremely poor housed in Worcester, Massachusetts.	Chronic pain.	Not specific (bodily pain).	Used the Pain Scale of the Short-Form Health Survey to detect pain and functional limitations due to pain in the past four weeks. Responses below the standardized score of 60 were considered to have physical limitations due to pain.	220 (homeless mothers); 216 (low-income mothers)
Bauer 2016	United States	Other: Retrospective Record Review	Drug overdose decedents identified in a prior analysis of mortality among adults who were seen at BHCHP between January 1, 2003, and December 31, 2008.	Chronic pain.	Not specific (Chronic pain and it's co-morbidity with conditions such as problem substance use, psychiatric illness, and medical conditions).	Self-reported chronic pain.	219
Bennett 2019	United States	Cross sectional study	Military veterans who served during the Iraq and Afghanistan conflict era, post 9/11, and reported any licit or illicit opioid use within the 30 days prior to enrollment. Recruited throughout NYC using venue-based and chain-referral sampling.	Not specified.	Not specific (overdose risk-behaviour and association with pain severity/interference, mental health difficulties, and stressful major life events).	Reported general pain severity and pain interference in the last 30 days.	218
Black 2013	United States	Cross sectional study	Adult patients from 540 treatment facilities in 410 three-digit ZIP codes in 35 states across the U.S., who reported past 30-day abuse of any prescription opioid on the ASI-MV assessment.	Not specified.	Not specific (pain problem).	A series of questions within the ASI-MV included questions regarding using prescription opioid for pain as well as the indicators of utilizing medical system, which includes recently received treatment for pain problem.	29,459
Chatterjee 2018	United States	Qualitative research	Are part of a family unit, living in Massachusetts, experiencing homelessness and OUD, and receiving care from Boston Health Care for the Homeless Program Family Team from January 2015 to August 2017.	Chronic pain.	Back injury, dental pain, and pain from a previous caesarean section.	Reported any physical pain, including chronic pain, that lead to prescription drug use.	14
Craig 2020	Canada	Literature Review	Socially marginalized populations: People who are indigenous, recent immigrants or refugees, of colour, LGBTQ2S, less well educated, living with mental health or substance-use challenges, or have experienced violence and trauma.	Chronic pain.	Not specific.	Literature review on the characteristics and aspects of pain in socially marginalized population.	N/A
Creech 2015	United States	Cross sectional study	Homeless Veterans presenting to a Homeless Patient Aligned Care Team program based at the VA Hospital	Chronic pain.	Joint pain disorder and chronic pain disorder.	Current chronic pain disorder (such as headache or migraine, joint pain disorder such as arthritis).	352
Dahlman 2017	United States	Cross sectional study	People who inject drugs in San Francisco (who are 18+).	Chronic pain.	Lower limbs, back, abdomen/genitals, neck/shoulders, head, and upper limbs.	Multivariate logistic regression analysis was conducted to examine the associations among self-reported pain dimensions (past 24-h average pain, pain interference with functional domains) and NMPOU, controlling for age, sex, psychiatric illness, opioid substitution treatment, homelessness, street heroin use and unmet healthcare needs.	702
Dworkin 1994	United States	Systematic review	Individuals with Schizophrenia	Acute and chronic pain.	Not specified (discussion on pain insensitivity).	Pain insensitivity associated with Schizophrenia, reported as both acute and chronic pain.	N/A
Flanagan 2016	United States	Qualitative research	from Community Shelter and Services (CSS) and The Welcoming Place (WP), which, at the time of the study, were homeless service entities in metropolitan Atlanta. They provided a variety of services for homeless former drug users in Atlanta. Twelve local experts in addiction agreed to participate, where: 8 were formerly homeless individuals recovering from substance abuse (4 African American men, 3 white men, and 1 white woman); 2 medical doctors with experience caring for persons who are homeless; 2 leaders of homeless advocacy groups, including a copartner of the WP and executive director of the CSS.	Chronic pain.	Not specific (significant physical pain).	Ethnography of homeless recovery from drug use in Atlanta, Georgia. Conducted participant observation, unobstructive observation, and in-depth interviews.	12
Fond 2019	Other: France	Randomised controlled trial	Recruited by mobile mental health outreach team. Participants were homeless adults, who were either diagnosed as Bipolar or schizophrenic patients	Chronic pain.	Not specific (physical pain).	Self-reported physical pain and its association to more frequent use of anti-depressants, identifying as female (gender), bipolar disorder, older age, and a higher MCSI psychotic score. Regardless of the reported number of days in the last 180 days participants reported being in the streets, MCSI depression score, both alcohol and substance use disorders, and psychoactive and analgesic drugs.	655
Gabrielian 2016	United States	Cross sectional study	VA Greater Lost Angeles’ supported housing enrollees	Chronic pain.	Either generalized or localized to the back or a joint.	Chronic pain and association with premature exit from supported housing before final house placement.	102
Gilmer 2020	Canada	Other: Narrative interviews (Qualitative data) as well as reviews of visit history record of local hospitals.	Canadian-born, self-identified as homeless or vulnerably housed in 2 rural and semirural towns in Ontario.	Chronic pain.	Not specific (Self-reported pain).	Reported pain and connection to discrimination and clinician bias due to social inequalities.	53
Golub 2013	United States	Non-randomised experimental study	OEF/OIF veterans returning to low-income predominately minority sections of NYC. Were recruited using Respondent-Driven Sampling as well as snowball sampling.	Not specified.	Not specific (self-reported pain).	Reported pain and the pathways to misuse of painkillers; recreational, iatrogenic, and opportunistic.	269
Gundlapalli 2017	United States	Cross sectional study	Homeless and non-homeless Veterans who used ED/urgent care clinic at 152 hospitals that are affiliated with the US Department of Veterans Affairs around the US.	Not specified.	Non-Homeless: Unspecified chest pain, abdominal pain, headache, other chest pain, Homeless or with housing instability: chest pain, pain in limb, abdominal pain.	Examining both homeless and non-homeless veterans and their differing uses of the ED. Homeless veterans more often reported health related concerns about alcohol dependence, depression, and alcohol abuse, not significantly associated with pain.	1,018,741
Hansen 2011	United States	Cross sectional study	A high-risk community-based cohort of HIV-infected indigent adults who had high rates of chronic non-malignant pain and illicit substance use and resided in an area known for the availability of nonmedical prescription opioids in San Francisco.	Chronic pain.	Not specific (severe pain).	Self-reported severe pain (chronic pain) and it's association with aberrant behaviours that can lead to mistreatment of pain.	296
Harris 2020	UK	Other: convergent mixed methods	Adults with history of injecting drug use who were predominately male, of White ethnicity, and a mean age of 46 years. The primary drug injected was heroin, either alone or with crack cocaine. High lifetime history of street homelessness, with less than half reporting current precarious housing. Most had experienced an SSTI, where a about half had hospitalization. Most sought medical care for an SSTI with the majority primarily using accident and emergency hospital services.	Chronic pain.	Not specific (severe physical pain associated with injecting-related injuries).	Normalized (severe physical) pain and its connection to avoidance of seeking out treatments due to marginalization.	455 for survey sample, and 36 for qualitative interview
Humphreys 2018	United States	Cohort study	Adults aged 55 or older enrolled in an urban county jail who used ED	Chronic pain.	Not specific (any pain in reported in the study apart from smaller injuries such as headaches, sprains, or toothaches).	Reported pain and the multimorbidity's associated with older age inmates.	101
Hwang 2011	Canada	Cross sectional study	Individuals who are 18 years or older were recruited at 17 shelters for single homeless adults in Toronto.	Chronic pain.	Back pain, knee pain, shoulder pain, foot pain, ankle pain, abdomen/genitourinary pain, neck pain, other locations.	The Chronic Pain Grade questionnaire was used to classify participants according to their overall pain severity. This measures chronic pain severity in three dimensions: intensity, disability, and duration. Information on the participants' chronic pain history, including duration, location, and the cause of pain, was also collected. Participants were asked about their use of treatments for their pain in the past three months. Additionally, obtained information on chronic pain management barriers. Participants were also asked to describe the frequency of drug and alcohol use as well as their perceptions on the effect of the drug on their pain as part of examining concurrent medical conditions.	146
Ito 2014	Other: Japan	Cross sectional study	Homeless persons from two areas of Tokyo, who experienced marginal homelessness, during the study period, and received support from a non-profit organization that offered support to homeless peoples in the survey areas.	Not specified.	Not specific (pain).	Pain and the association with low well-being of homeless individuals in Japan.	423
Johnson 2013	United States	Other: Literature Review	US military veterans, including a significant number of wartime veterans, who had more specific health issues particularly on traumatic brain injury, polytrauma, hazardous exposures, chronic pain, posttraumatic stress disorder, military sexual trauma, substance use disorders, suicide, and homelessness.	Chronic pain.	Not specific.	The various impacts, such as chronic pain, that plague US military veterans and their health, and suggestions to combat such health hindering factors.	N/A
Johnstone 1994	United States	Other: Descriptive study	CHP homeless clients who had received Western-style medical evaluation and treatment prior to receiving acupuncture.	Not specified.	Arthritis (unspecified), back pain, headache, abdominal pain.	The use of acupuncture to alleviate reported pain in the homeless population.	45 for part 1, and 30 for part 2
Jones 2016	United States	Other: mixed-methods design	Community and Veterans Affairs Leaders and homeless veterans who were recruited from southern, northeastern, northwestern, and southwestern regions of the US.	Chronic pain.	Not specific.	Managing chronic pain in the context of palliative care for homeless individuals with comorbid mental health issues.	136
Kip 2016	United States	Cohort study	Homeless veterans who had temporary residence of HEP and veterans who were treated at community-based sites.	Not specified.	Not specific (general pain associated with trauma).	Testing the effectiveness of an Accelerated Resolution Therapy, which focuses on treating trauma symptoms, and the association of such trauma with pain.	140
Kneipp 2015	United States	Systematic review	Disadvantaged women who experienced migraine headaches.	Chronic and acute.	Headache.	Migraine pain and its various associations, such as comorbid depression specifically in the disadvantaged, self-identifying, female population.	N/A
Landefeld 2017	United States	Cross sectional study	Homeless adults who resided in overnight shelters, homeless encampments, meal programs, and a recycling centre in Oakland California who were 50 years or older.	Chronic pain.	Not specific.	Factors associated with reporting moderate, severe, or chronic pain in the aging homeless population, such as previous abuse and PTSD.	350
Lankenau 2012	United States	Other: mixed method (ethno-epidemiological methodology)	Young injection drug users (aged between 16 and 25) who had misused prescription drugs at least three times in the past three months in LA and NY and were both homeless and transient.	Short-term, acute, and chronic pain.	Not specific (untreated pain).	Young, homeless injection drug users that self-medicate for untreated chronic pain, and various drug use dependencies.	50
MacNeil 2011	Canada	Qualitative research	People who used injection drugs and were using needle exchange services throughout the region. Majority of the sample were homeless or living in precarious housing, on financial assistance, and had been local residents for 5 years or more as well as being in the area for more than a decade. The majority were polydrug users.	Not specified.	Not specific.	Pain and its relation to initial drug use as a coping mechanism, but how it can morph into a non-helpful problem as well as a drug use problem.	33
Matter 2009	United States	Other: cognitive interview method	Homeless individuals at Seattle area shelters, transitional housing programs, and public agencies. Majority were white, male, unemployed or disabled, and reported some post-high school education. Most have been at shelter/transitional housing/tent city for at least one night over the past three months. Most reported health arthritis, where nearly half of the participants reported their average level of pain and fatigue over the past week as severe or very severe, while the remainder reported mild to moderate pain and fatigue.	Chronic pain.	Arthritis, back pain, multiple sclerosis pain, pain associated with both AIDS and lupus.	Examination of the unique experiences of homeless individuals who report pain, such as how weather and sleeping conditions can affect pain. In addition, the creation of measures that accurately distill the experience of homeless individuals with pain.	17
McNeil 2012	Canada	Qualitative research	Health and social services professionals (including physicians, health administrators, nurses, shelter directors, harm reduction specialists, outreach workers, and personal support workers) involved in the delivery of health and end-of-life care services to homeless persons who use illicit drugs in Halifax, Ottawa, Toronto, Thunder Bay, and Winnipeg.	Not specified.	Not specific (general pain management).	Barriers and faulty understanding on how to safely manage pain in the homeless population. Factors include mistrust in the healthcare system/patrons, not wanting to disclose substance use.	50
Miaskowski 2011	United States	Cross sectional study	HIV-positive adults who resided in San Francisco, were socioeconomically disadvantaged, and were recruited from homeless shelters, free-meal programs, and single-room occupancy hotels.	Chronic pain.	Calf pain, foot pain, and lower back pain.	Homeless individuals with HIV living with varying levels of pain, with most participants reporting higher levels to chronic pain. Examining various factors associated with pain, such as female (gender) and less education, as well as depression.	296
Nyamathi 2012	United States	Randomised controlled trial	methamphetamine, cocaine, and crack-using G/B young adults who frequented a community site in Hollywood, California.	Chronic pain.	Not specific (body pain).	Depressed mood in a sample of young stimulant using gay and bisexual homeless men and its association with reporting severe bodily pain.	267
Okamura 2014	Other: Japan	Cross sectional study	Homeless people who resided on streets or riversides or in urban parks or stations (street homelessness), or residents of shelters, cheap hotels, and welfare homes for homeless people (sheltered homelessness) in two districts of Tokyo, Japan.	Not specified.	Not specific (general pain).	Statistical significance with a sample of homeless individuals residing in Japan, who report suicidal ideation and high levels of pain.	423
Painter 2018	United States	Other: Retrospective Data Review	Veterans’ Health Administration patients with substance-use disorders alone or with co-occurring mental health conditions.	Chronic pain.	Not specific.	Predictors of high inpatient utilization included homelessness, pain, co-occurring mental health issues, etc.	470,548
Pangarkar 2021	United States	Systematic review	Homeless population experiencing pain.	Acute and chronic pain.	Not specific (bodily pain).	Review of literatures on various aspects and elements of pain among homeless population.	N/A
Paudyal 2021	UK	Other: Analysis of routinely collected data set	Persons experiencing homelessness who were often excluded from primary health care and community prevention programmes leading to high use of hospital emergency departments in West Midlands region of England.	Not specified.	Not specific (a single pain category).	Inappropriate drug use and pain were associated with presentation at the ED, and pain was more than twice as likely to be reported from females than males.	3,271
Paul 2020	Canada	Qualitative research	Vulnerable young people who experienced street entrenchment using substances. Also, youth-focused care providers including GP, NP, nurse, drug alcohol counselors, and social workers.	Acute and chronic pain.	Back pain and stomach pain.	Cannabis use and it's therapeutic, pain-inhibitory properties for street-entrenched youth.	56 young people and 12 youth-focused care providers.
Poleshuck 2006	United States	Cross sectional study	Low income, primarily African American women presenting at an urban women's health clinic for routine gynaecological care.	Chronic pain.	Back pain, neck pain, headache or migraine, stomach-ache, or abdominal pain, gynecological or pelvic pain, joint pain, chest pain, facial ache or pain.	Used the Graded Chronic Pain Scale to measure pain. Also used Short-Form Health Survey, which includes questions specifically about bodily pain.	242
Raven 2017	United States	Cross sectional study	A sample of adults aged 50 or older, on Oakland, California, who met criteria for homelessness.	Chronic pain.	Not specific (severity of pain: mild/moderate and severe) and oral pain.	Pain and its association with higher rates of using the ED in a homeless sample, due to it being a low-barrier option to seek treatment.	350
Reuven 2021	United States	Cross sectional study	Adults, at the time, experiencing homelessness, who were recruited from six homeless-serving agencies in Oklahoma City.	Acute and chronic pain.	Not specific (severity of pain).	The mediating effect of Anxiety Sensitivity Treatment on pain and smoking in a sample of homeless.	610
Riley 2003	United States	Cross sectional study	HIV-positive homeless and marginally housed individuals in San Francisco.	Not specified.	Not specific (bodily pain).	Health questionnaires administered included SF-36, where one of the eight scales is Bodily Pain.	330
Ronksley 2016	Canada	Other: Retrospective Observational study	Adults with at least three visits to the Ottawa Hospital ED within a one-year period.	Chronic pain.	Abdominal pain, chest pain, lower extremity pain, back pain, pelvic pain, throat pain.	High use of the ED but in a short period of time, were more likely to be homeless, to leave before being properly treated, and to return for the same problems (most often a pain disorder).	16,153
Rudolph 2020	United States	Randomised controlled trial	English-speaking adults with DSM 5 OUD.	Not specified.	Not specific (self-reported pain).	Goal was to map out relapse likelihood of two groups, homeless and non-homeless who both were diagnosed with opioid use disorder, using both extended-release naltrexone versus buprenorphine-naloxone. Pain was assessed on the day of the assessment. Participants randomized to medication. Pain was assessed each week of assessment using a version of EuroQOL questions that asked participants to define their pain on the day of the assessment. Scores then were averaged after treatment and prior to date of relapse/dropout.	570
Salem 2019	United States	Cross sectional study	Homeless, female, ex-offenders enrolled in a larger interventional study, which randomized participants into one of two programs aimed at reducing drug use and recidivism.	Not specified.	Not specific (bodily pain).	Within a sampled female (gender) ex offender, associations with physical frailty includes factors such as years experiencing homelessness, observing less violence, drug dependence, and PTSD. Whereas with psychological frailty some factors included time spent homeless, pain, and drug use/dependence.	130
Savage 2006	United States	Cross sectional study	Homeless persons who utilized the nurse-managed clinic.	Chronic pain.	Back pain.	Examining various needs of homeless adults that utilized a nurse-managed clinic. Most health diagnoses were of hypertension, arthritis, asthma, and chronic back pain. Not much differed between those who did use the ED and those who did not, besides insurance.	110
Seto 2021	United States	Case control study	Homeless individuals who presented with rheumatic and/or musculoskeletal disease that were being seen at the L.A. County Medical Center of the Keck School of Medicine of the University of Southern California.	Chronic pain.	Joint pain.	Detailing the unique and significantly worse health outcomes of homeless individuals with rheumatic and musculoskeletal disorders. One of the biggest issues was the access to healthcare and medicine, utilizing emergency service more often, and displaying alarming disease progression, often with high levels of pain that impeded daily activities.	34
Sturman 2020	Australia	Qualitative research	Male clients of an 80-bed Brisbane homeless men's hostel.	Not specified.	Not specific (description of the difficulty homeless individuals have navigating the healthcare system).	The healthcare system for homeless individuals appears to be more negative than beneficial. Some of the factors that lead to homeless individuals having more difficulty navigating the healthcare system are dismissive care, care fragmentation, variability in terms of the management of pain, and inadequate acknowledgement of psychological distress on part of healthcare workers.	17
Tobey 2017	United States	Other: Survey	Homeless individuals at a 104-bed medical respite program for homeless, who were at or near the end of life.	Not specified.	Not specific (examining end of life and its association with pain).	Homeless individuals facing the end of their lives reported high symptomatic levels of pain and various psychological effects.	20
Tong 2019	United States	Cross sectional study	50 years or older individuals who, at the time of the study, were experiencing homelessness and food insecurity in Oakland, California.	Chronic pain.	Oral pain.	Examining the aging homeless population and reporting symptoms associated with food insecurity, which is common in this population. Common symptoms include depressive symptoms, oral pain, and cognitive impairment.	350
Usherwood 1993	UK	Cross sectional study	Hostel residents who were homeless in an inner-city area of Sheffield.	Not specified.	Not specific (general pain).	Using the SF-36 to interview individuals residing in a hostel about their current self-perceived health. Common themes found were that individuals who reported experiencing a current health difficulty scored lower on domains such as social function, physical role function, pain, and general health. Those who reported taking a form of prescribed medication also reported lower scores on pain, social function, and general health. Finally, those who had any hospital interaction within the prior three months had significantly lower scores in social functioning, emotional role functioning, pain, and general health.	104
Vogel 2017	Canada	Cross sectional study	Homeless persons with mental illness who were recruited from three Canadian cities, in the At Home/Chez Soi Study	Chronic pain.	Back pain, joint or limb pain, head, or neck pain.	Examining the relationship between mental health and pain in the homeless population. Such pain disrupts activities such as daily mundane activities, sleep, and social interactions. In this study, predictors of experiencing high levels of pain were increasing age, being diagnosed with major depressive disorder, mood disorder with psychotic features, panic disorder, and PTSD, it also was associated with a higher level of suicidality.	1,287
Wan 2006	United States	Cohort study	Patients admitted to an urban Level I trauma center with unintentional injury.	Not specified.	Not specific (unintentional injury and pain insensitivity).	Used databases (the San Francisco General Hospital Trauma registry, the hospital's medical record, and the San Francisco Department of Public Health Billing Information System) for statistical examination. The researchers linked high rate of unintentional injury admissions among mentally ill patients to decreased pain sensitivity in those with mental illness.	1,709
Weinreb 1998	United States	Case control study	Homeless mothers and low-income housed mothers who were receiving welfare.	Chronic pain.	Not specific (bodily pain).	Investigating both homeless women and low-income housed women, who would be deemed the 'head of household', and their unique health-based concerns. Both groups of mothers experienced low levels of role and physical functioning and higher levels of bodily pain.	416 (220 homeless mothers and 216 low-income housed mothers receiving welfare)
Wenzel 2006	United States	Other: comparative research	Young women from shelters and Housing and Urban Development Section 8 low-income housing in LA County.	Chronic pain.	Pelvic pain, back pain, upset stomach, severe headaches/migraines.	Understanding and highlighting both the physical and sexual violence that many younger indigent women face and its association with physical and behavioural health. The young women, recruited from various shelters, who had experienced past or present physical and/or sexual violence were more likely to present with an STD, vaginal discharge or bleeding, and pelvic pain in the past 6 months. Within 12 months, they were more likely to be diagnosed with drug abuse or dependence and depression.	224
Witham 2019	UK	Other: Rapid Evidence Assessment	Papers about people who used alcohol and other drugs, were over the age of 40, are likely to be in greater need to access palliative and end of life care services.	Not specified.	Not specific (pain management).	Analyzing various papers on the distinctive needs of those who have alcohol and drug problems, with respect to their end-of-life care. Suggesting that when it comes to individuals who are both homeless and have substance use problems, safe prescribing of pain management needs to create a more flexible model to identify Sneeds and find an appropriate way to address such issues.	60 papers
Wolford-Clevenger 2019	United States	Cross sectional study	Women who resided in a domestic violence shelter, at the time of the study, due to intimate partner violence.	Not specified.	Not specific (pain tolerance).	Examining women in shelters that are seeking refuge from intimate partner violence. The authors used interpersonal-psychological theory of suicide (IPTS), which states that suicidal ideation is more likely to come about from hopelessness about feelings of not belonging and personally believing one is a burden, but caveats that this cannot occur unless one has acquired the capability for suicide (i.e., pain tolerance and fearlessness about death). However, the only significant association was that physical partner violence was associated with capability for suicide.	134

All literature contained investigation of mental illness or psychiatric conditions interacting with pain in unique ways. Most of the studies examined mental illness in a broad sense. The specific psychiatric conditions that were analyzed were as follows: a large proportion of the studies assessed substance use dependencies (7, 15, 16, 19, 30–44); PTSD (16, 18, 45–47); bipolar disorder (33); schizophrenia (48) and (33); depressive disorders (18, 30, 33, 39, 43, 49–53).

All studies examined pain, in differing significance, with the vast majority examining reported chronic pain. Specific examinations of pain in the various studies included pelvic pain reported from indigent women in (43), oral pain (52), headache or migraine pain (54, 49), and finally, inflammatory diseases, associated with pain, such as arthritis (54–57).

### Population characteristics

In all the studies examined, the age range of participants was 19–95, with the average age roughly estimated in their 40's. Thirty nine out of 57 (68.4%) of the selected studies had a majority representation of males, 7/57 (12.3%) had solely female representation and 1/57 (0.02%) had a majority representation of females. Ten out of 57 (17.5%) did not specify gender as they were limited due to their study design (e.g., narrative review) and the questions they set out to answer. Ethnicity varied substantially across the studies, with 17/57 studies not specifying ethnicity. Twenty one out of 57 studies had a majority of Caucasian participants, 16/57 had a larger proportion of African American/Black participants, and individually Bassuk et al. (45) identified race as non-white making up 66% of their sample; Okamura et al. (51) examined individuals of Asian heritage, constituting all of their sample, and Weinreb et al. (58) studied those identifying as Puerto Rican.

Due to the complex scope of our review, and the limited literature on mental illness and reported pain in the homeless population, it is important to note that it is not possible to provide full specifics on each population studied. As anticipated, we found that the homeless populations were non-homogeneous, and there was not a common denominator for the many different cohorts that had been studied. Further descriptors to note were that 8/57 studies included homeless veterans; 37/57 studies included discourse on medical treatment and observation; 6/57 studies spoke on the unique experience of homeless women experiencing pain with regards to motherhood, sexual and physical violence, and the unique pain they experience; and finally, 2/57 studies examined homeless individuals in jail or ex-offenders.

### Factors in aggravation of reporting pain in the homeless population with concurrent psychiatric conditions

Precarious housing, including homelessness, is associated with high rates of pain (4, 8, 46, 52, 55, 57, 59–61); for example, one observational study reported that 63% of homeless subjects endorsed experiencing pain lasting longer than three months (62). Living with pain disrupts securing housing (7, 16), and in turn precarious housing also has a significant impact across a wide spectrum of variables on the person experiencing pain. While living with psychiatric condition(s) alone can lead to many challenges in performing daily tasks, experiencing pain may be a significant additional stressor and add further impairment; for example, a study of 1,204 subjects with chronic disabling pain that was unresponsive to medical treatment noted that those with concurrent depression were “more likely to be unable to work because of ill health and reported greater work absence, greater pain-related interference with functioning, lower pain acceptance, and more generalised pain” compared to those without concurrent mental illness (63). Dworkin (48) examined pain insensitivity among people with schizophrenia, where this population reported significantly reduced pain sensitivity while experiencing various severe medical conditions including third-degree burns, cancer, and heart diseases at a rate presumably higher than in the general population. Substance use disorder is also related to chronic pain in various ways. For instance, it was noted that some individuals started using prescription opioids due to chronic pain [in contrast to the role of non-prescription opioids, which we recently examined in detail in the homeless population (4)]. Worsening pain, including pain from drug withdrawal or conditions such as chronic arthritis, then led to additional problems when co-occurring with an existing psychiatric condition, such as getting into financial difficulties, a worsening of housing-related problems, interpersonal problems, as well as struggles with taking care of family and other aspects of daily life (7, 16, 17, 19, 42, 64). Such challenges then also then led to disruptions in receiving appropriate treatment. For example, Opioid Use Disorder treatments can be difficult to implement effectively due to financial issues, transportation issues, associated stigma, the emotionally challenges involved with treatment, and interference in various other everyday tasks (7, 65). Thus, there is a high prevalence of reporting pain among people with substance use disorder (7, 16, 19, 35, 42, 46, 66), as well as with low mental well-being (61)—for example, it was reported that 37%–60% of patients in methadone maintenance treatment for opioid dependence exhibit concurrent chronic pain (67, 68). A large number of people experiencing pain also co-reported Posttraumatic Stress Disorder (PTSD) (7, 16, 35, 43, 47, 66), as well as depression and anxiety (43, 47, 64–66).

The quality of the experience while receiving medical treatment is important for patients; however, negative interactions with healthcare professionals can play a role in under-reporting pain. Possible discrimination as well as stigma associated with the healthcare system was found to be one of the main barriers to effective treatment in multiple studies (7, 18–20, 36, 53, 60). Additionally, physicians may be unwilling to prescribe pain medication due to patients' substance use history, psychiatric condition(s), and lack of compliance (17). Attempts to self-medicate pain or other health problems due to the barriers mentioned above can lead some to use illicit drugs (17, 19, 60, 69), which then puts them in situations where they face even more challenges, potentially resulting in greater social withdrawal (49). Thus, when the primary problem with pain is not effectively resolved, it may result in an exacerbation of that pain leading to further challenges and worsening of concurrent psychiatric conditions (4).

### Examples of pain management with positive responses in homeless population

It is informative to describe in some detail the different approaches to pain management in the homeless and precariously housed population, such as (CBD) pills, acupuncture, and accelerated resolution therapy (ART), which examines individual's trauma to address pain, as well as depression (see below). Evidently, the management of pain within this population requires treatment that is tailored to their specific circumstances. When addressing such chronic/acute pains, it is important to think about the circumstances that these individuals find themselves in currently, in respect to their living, their food consumption, their personal healthcare options, and have previously endured, such as traumatic events including sexual assault, being a war veteran, and family abuse. Various treatment methods have been studied to suggest potential options, and below we discuss three experimental options.

A number of participants reported that using cannabis was more effective and “healthier” than the traditional psychopharmaceuticals and medication assisted substance use treatment (41). While cannabis provides a hedonic and pleasurable experience, and has been reported to help with chronic neuropathic pain (70–72), study participants noted improvement not just in chronic pain but also anxiety, depression, and attention deficit hyperactivity disorder (ADHD) (41). Cannabis is also seen as a form of harm reduction by some because it lessens the severity of withdrawal, while others see it as a form of treatment that helps them not only with the easing withdrawal symptoms, but also with avoiding relapse (41).

Among veterans in homeless shelters suffering from PTSD, the non-pharmacological treatment Accelerated Resolution Therapy (ART) used in treating trauma also showed improvement in symptoms of pain as well as depression, anxiety, and quality of life (47). While the improvement of symptoms was observed among those who completed ART, Kip et al. (47) acknowledged the rate of completion was low among homeless veterans when compared to their counterparts. Thus, such treatments will benefit from the development of more effective protocols to ensure treatment is completed (17, 47).

Homeless persons receiving acupuncture treatment at the Chicago Health Outreach Clinic to ease arthritis, headache, back pain, substance abuse, anxiety, fatigue, and other diagnoses reported favourable responses overall, where part of the study recorded a 97% positive response (73). The study found that even those who previously did not see much improvement or no improvement from Western medical treatments saw positive results (73). Additionally, acupuncture is cost effective with fewer severe potential side-effects compared to opioid therapy (73). Although the two-part study had a small sample size and was neither controlled nor blinded, the findings suggest that alternatives to traditional pain treatment can be studied with better designed trials to address pain and other associated comorbidities.

## Discussion

This review summarized findings from articles examining the associations between pain and mental illness within the homeless population. The detailed literature was surveyed for both pain and mental illness, while examining the nature of the relationship between these two across multiple different domains. This review set out to explore the common concurrent psychiatric conditions that are associated with chronic pain in the homeless population, and furthermore, delved into factors that could aggravate the act of reporting pain in the homeless population with concurrent psychiatric conditions. The main findings from this review highlight the unique and complex difficulties presented with this population.

The literature shows that a history of stress, abuse and trauma is important to consider when examining pain. Throughout the literature, there is considerable uncertainty surrounding whether drug use precedes or follows pain, and it is evident that both scenarios do transpire. In addition, housing issues that come with being homeless, such as the vulnerability to the elements, potential violent/sexual encounters, overcrowding in shelters, the requirement to trek long distances, not having proper bedding, and many other factors have been found to worsen ill health and exacerbate pain. Furthermore, suboptimal treatment from the healthcare system has also been found to aggravate pain within this population, and prevent them from seeking out the proper care that is needed. These issues in the healthcare system can prevent individuals from receiving the appropriate care for pain as well as for psychiatric conditions. This often leads to avoidance of seeking various needed treatments and using social withdrawal as a way of coping with pain. Other significant factors include the cost of treatments, and the transportation that is often not available.

In addition to standard medications for pain relief, a small number of alternative pain therapies had been tested in the homeless and marginally housed population, although issues around experimental design, samples size and recruitment will make future studies challenging. Cannabis use was self-reported by some as a more effective and amenable substitute to other more conventional routes such as psychopharmaceuticals and medication-assisted substance use treatments. The use of acupuncture to alleviate self-reported pain within the homeless population showed a positive response. Finally, Accelerated Resolution Therapy, which bases its practice in treating trauma symptoms and the pain that is associated with said trauma (47), showed potential. However, as noted above, it is evident that there are barriers present in today's current social climate that restrict the homeless population from accessing treatments such as these to address concerns and feelings of pain.

### Recommendations

1. Opioid abuse is closely associated with chronic pain related issues than other types of substance use. Therefore, harm reduction programs, such as safe consumption or injection sites, should become more available and accessible. Such initiatives should then help with minimizing potential harm from substance use, especially with opioid use, in the management of higher levels of pain.
2. Stable residence.
  a. We would like to emphasize the importance of reliable and stable housing as the base of the whole treatment picture. There is an imminent need for those who present as having psychiatric needs, dealing with disabilities, are elderly, and/or are single to have affordable housing. This housing should employ competent support workers who can demonstrate thorough understanding of the unique needs of and circumstances of the cohort they are taking care of.
3. Access to support groups that are led by a qualified and licenced professional or physician would be greatly helpful in alleviating this biopsychosocial issue of pain.
4. Improvement on stigma around homeless and precariously housed individuals with psychiatric illness and pain issues among the public as well as health professionals would allow better access to health care and appropriate treatments. Raising awareness among the public about the issues that are experienced by this cohort without labeling or stereotyping, but rather with the view that they are individuals with health issues like everyone else, would improve the quality and availability of treatments. The increased awareness would also have potential to inform policy decisions and allocation of funding.
5. More research should be conducted in treating this cohort in a holistic biopsychosocial approach, attending to the unique and specific needs and circumstances, as suggested by various studies including those examined in our review. Even if we see positive results from a treatment developed for a general population, more effort to tailor treatments and approaches is needed to maximize positive outcomes in this cohort.

### Limitations

Generally, there is a paucity of research that examines mental illness and pain in the homeless population, given the numbers of individuals involved, their medical complexity, and the medical resources required to address this issue. A limitation of this review includes the predominance of literature collected from North American studies, and in turn with that, our inability to include studies not published in English due to a language barrier. To overcome this limitation, our team, if greater funding were available, we could have incorporated studies printed in other languages translated into English to ensure we were obtaining a more comprehensive view of these issues across the planet. As an extension of this issue, there are considerable differences in healthcare systems and clinical resources within North America alone—based on country, state/province, and other geographic variables such as urban vs. rural. These differences make generalized inferences about treatment deficiencies for this population challenging, and recommendations for improvement are likewise difficult. While we had initially planned to use a common concept of pain when we initially started searches for literature (such as pain scores on standardized pain questionnaires), it was quickly apparent that most studies use a much more diverse set of reporting conditions when describing pain and comorbid mental illness in the homeless population. This lack of standardization means that in reviewing the literature, major themes emerge but commonalities tend to be less granular in detail across the field; thus, our review was required to examine pain in varied ways due to the diverse and unique types of pain presented within these studies. As a caveat, Matter et al. (60) noted that appropriate modifications should be applied when conducting cognitive interview processes in measuring pain among the homeless population. The majority of studies we reviewed, however, utilised pain measurement metrics that are designed for the general population, not for this specific population. With respect to bias, we have been attentive to the application of certain parts of the systematic review process and recognize the possibility for subjectivity.

**Figure 1:**
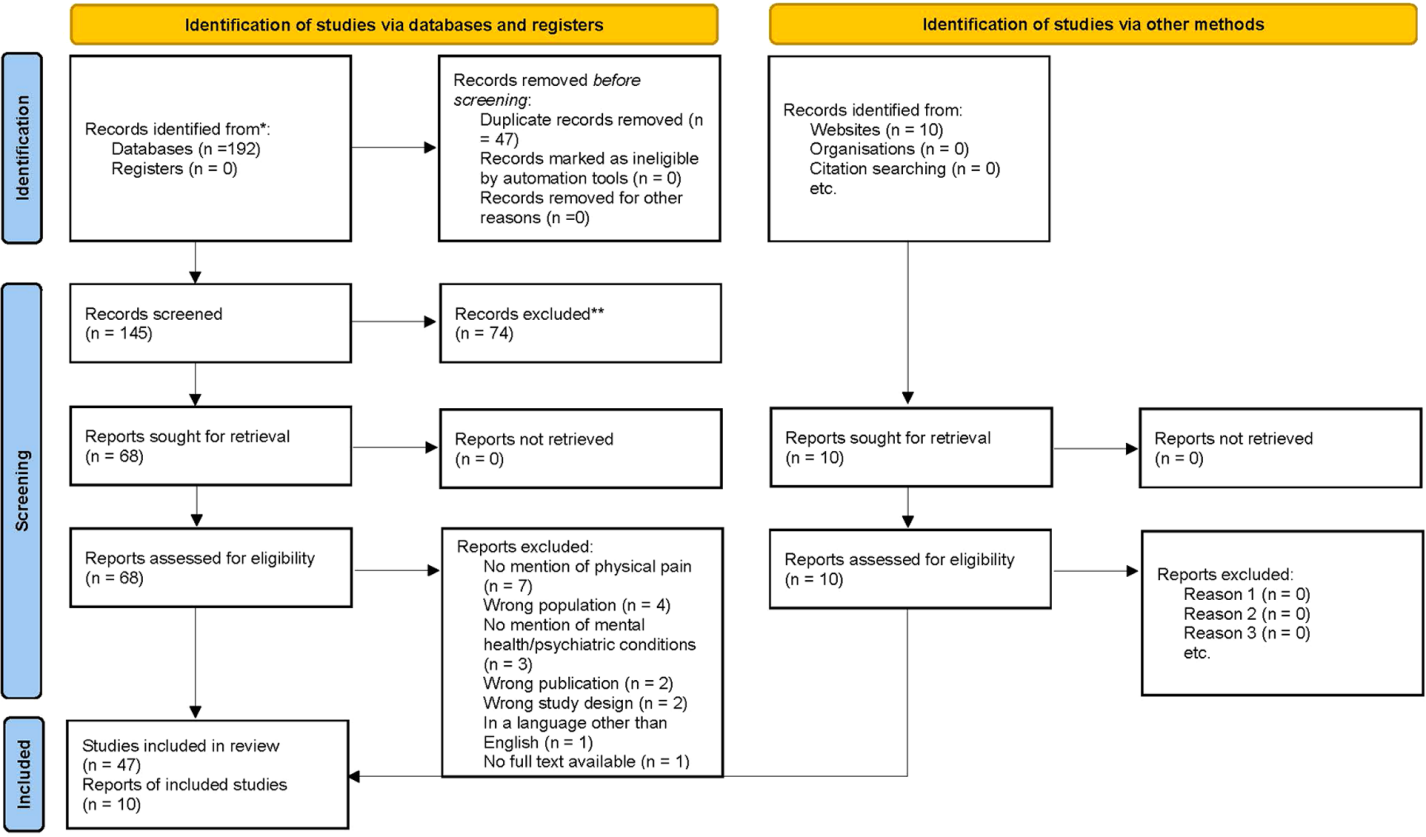
PRISMA 2020 flow diagram for new systematic reviews which included searches of databases, registers and other sources. Citation: From: Page MJ, McKenzie JE, Bossuyt PM, Boutron I, Hoffmann TC, Mulrow CD, et al. The PRISMA 2020 statement: an updated guideline for reporting systematic reviews. BMJ 2021;372:n71. doi: 10.1136/bmj.n71.

## Conclusion

This scoping review highlights and further cements housing status as an important factor when considering an individual's current health status and the further impact housing has towards a person's health. It has become increasingly clear that the unique health determinants that this population displays have contributed toward, and is a symptom of, a widespread pandemic among people who are homeless. Due to the disparate nature of the services available for this community of peoples, it has become clear that there is not currently a comprehensive way to adequately address the unique treatment requirements of this population. With this in consideration, this results in the exacerbation of the chronic pain that is commonly experienced within this population. In the variety of support services, there needs to be enhanced training within those positions, to minimize stigma that can adversely affect and exacerbate this population's unique levels of pain in conjunction with mental illness. Additionally, healthcare workers who interact with this population, should consider gaining beneficial knowledge on pain management, in conjunction with a comprehensive education on mental health. This should also include training in integrated treatment (74), which ensures that treatment is straightforward, co-ordinated and comprehensive for the client. Integrated treatment also ensures that the client is provided with assistance not only with the concurrent disorders, but importantly in additional day-to-day aspects, including housing and employment. Furthermore, if a client's treatment services are in multiple locations, the clinical services should work together to co-ordinate treatment; a recent systematic review reported that integrated models of clinical care for those with concurrent disorders are more effective than conventional, non-integrated models (75). With respect to shelters, a valuable addition would be to implement centralized information centres, with trained mental health and pain management professionals, to ensure correct information is being communicated to this particularly vulnerable population. Shelters could also benefit from inclusion of key services such as physical therapy, and technology to facilitate pain management tools.

With regards to research, better controlled clinical trials specifically for this population need to assess interventions for pain, and how this affects concurrent mental illness. These all represent significant challenges, but given the worldwide numbers of individuals living with both pain and mental illness in housing instability, a significant amount of human suffering could be reduced by improving outcomes in this population.

## Data availability statement

The original contributions presented in the study are included in the article/supplementary material, further inquiries can be directed to the corresponding author.

## Author contributions

KR reviewed abstracts, analyzed results and wrote the first draft of the review. ES assisted in abstract review. RMC helped with developing the Table. ENRS assisted with early stages of the systematic review formulation. AMB oversaw the project. All authors provided intellectual input into the final version of the systematic review and contributed to the final version of the manuscript. All authors contributed to the article and approved the submitted version.

## Conflict of interest

The authors declare that the research was conducted in the absence of any commercial or financial relationships that could be construed as a potential conflict of interest.
